# A new small-sized predatory pseudosuchian archosaur from the Middle-Late Triassic of Southern Brazil

**DOI:** 10.1038/s41598-024-63313-3

**Published:** 2024-06-20

**Authors:** Rodrigo T. Müller

**Affiliations:** 1 Centro de Apoio à Pesquisa Paleontológica da Quarta Colônia, Universidade Federal de Santa Maria, Rua Maximiliano Vizzotto, 598, São João do Polêsine, Rio Grande do Sul 97230-000 Brazil; 2 Programa de Pós-Graduação em Biodiversidade Animal, Universidade Federal de Santa Maria, Santa Maria, Rio Grande do Sul 97105-120 Brazil

**Keywords:** Palaeontology, Palaeontology, Phylogenetics, Taxonomy

## Abstract

Before the rise of dinosaurs and pterosaurs, pseudosuchians—reptiles from the crocodilian lineage—dominated the Triassic land ecosystems. This lineage diversified into several less inclusive clades, resulting in a wide ecomorphological diversity during the Middle and Late Triassic. Some giant pseudosuchians occupied the top of the trophic webs, while others developed extensive bony armor as a defense mechanism, which later evolved as a convergence in the avemetatarsalian lineage. On the other hand, there were groups like the Gracilisuchidae, which was composed of carnivorous forms with lightweight build and less than 1 m in length. The fossil record of gracilisuchids is geographically restricted to China and Argentina, with one ambiguous record from Brazil. In the present study, the first unambiguous gracilisuchid from Brazil is described. *Parvosuchus aurelioi* gen. et sp. nov. comes from the *Dinodontosaurus* Assemblage Zone of the Santa Maria Formation, which is associated with the Ladinian-Carnian boundary. Composed of a complete cranium, vertebrae, pelvic girdle and hindlimbs, the new species nests with *Gracilisuchus stipanicicorum* and *Maehary bonapartei* in a phylogenetic analysis. Its discovery fills a taxonomic gap in Brazilian pseudosuchian fauna and reveals the smallest known member of this clade from the *Dinodontosaurus* Assemblage Zone, highlighting the diversity of pseudosuchians during the moment that preceded the dawn of dinosaurs.

## Introduction

Shortly after the End-Permian extinction event^1^, the Triassic Period witnessed some of the most remarkable evolutionary episodes in the history of life, including the emergence of dinosaurs and pterosaurs^2–4^. However, reptiles from the crocodilian lineage—Pseudosuchia—ruled the land ecosystems before the dawn of the dinosaur age, achieving an impressive taxonomic and ecological diversity^5^. Some of the most impressive pseudosuchians from the Triassic Period were the giant quadrupedal apex predators, such as *Prestosuchus chiniquensis*^6,7^ and *Luperosuchus fractus*^8^. On the other hand, there were smaller faunivorous pseudosuchians that filled distinct niches, such as the lightly build gracilisuchids^9^. These small sized pseudosuchians were characterized by a relatively enlarged head with wide openings, carnivorous-like teeth, slender limbs, and a quadrupedal stance^10–14^. According to the fossil record, gracilisuchids lived from the Middle to the Late Triassic^15,16^. The oldest species is *Turfanosuchus dabanensis*^15^ and was recovered from the lower Kelamayi Formation of China, which is Anisian in age. A second species from China is *Yonghesuchus sangbiensis*^12^, which comes from the Upper part of Member II of the Tongchuan Formation, considered early Ladinian in age^17^. A putative coeval form comprises *Gracilisuchus stipanicicorum*^10^, from the Chañares Formation of Argentina^18^. Finally, a possible additional record from South America includes *Maehary bonapartei*^16^, which was excavated from the Caturrita Formation of Brazil (early Norian in age)^19^. This taxon was initially described as a pterosauromorph^16^. However, a recent phylogenetic study recovered it as a member of Gracilisuchidae^2^. If *Maehary bonapartei* is indeed a gracilisuchid, the fossil record of the group will extend to the early Norian. Despite this controversial record, no other specimen has been reported for Brazil^20^. This is particularly intriguing since the Triassic Brazilian beds are coeval with those in Argentina^18^, which have yielded records of gracilisuchids^10^. This gap is filled here with the description of the first unequivocal Brazilian member of this clade.

## Material and methods

### Institutional abbreviations

CAPPA/UFSM, Centro de Apoio à Pesquisa Paleontológica da Quarta Colônia da Universidade Federal de Santa Maria, São João do Polêsine, Rio Grande do Sul, Brazil; MCP, Museu de Ciências e Tecnologia PUCRS, Porto Alegre, Rio Grande do Sul, Brazil.

### Specimen

The specimen here described is housed at the Centro de Apoio à Pesquisa Paleontológica da Quarta Colônia/Universidade Federal de Santa Maria (CAPPA/UFSM), under the specimen number CAPPA/UFSM 0412.

### Phylogenetic analysis

The phylogenetic affinities of the new pseudouchian archosaur were investigated employing the data matrix of Müller et al.^2^. The analysis was performed in the software TNT v. 1.5^21^ following the parameters employed in the former study. Therefore, characters 9 and 119 were deactivated (these characters are non-independent from other characters added in previous iterations of this data matrix), all characters received the same weight, and characters 1, 2, 7, 10, 17, 19–21, 28, 29, 34, 36, 40, 42, 46, 50, 54, 66, 71, 74–76, 100, 122, 127, 146, 153, 156, 157, 171, 176, 177, 187, 202, 221, 227, 263, 266, 278, 279, 283, 324, 327, 331, 337, 342, 345, 351, 352, 354, 361, 365, 368, 370, 377, 379, 386, 387, 398, 410, 414, 424, 425, 430, 435, 446, 448, 454, 455, 458, 460, 463, 464, 470, 472, 478, 482, 483, 485, 489, 490, 502, 504, 510, 516, 520, 521, 529, 537, 546, 552, 556, 557, 567, 569, 571, 574, 581, 582, 588, 636, 648, 652, 662, 701, 731, 735, 737, 738, 743, 749, 766, 784, 803, 809, 810, 816, 850, 851, 872, 875, 885 and 888 were set as ordered. The final data matrix comprises 888 active characters and 215  active operational taxonomic units. *Petrolacosaurus kansensis* was used to root the most parsimonious trees (MPTs), which were recovered employing New Technology Search Algorithm, searching for a minimum length 100 times with the default Ratcheting, Drift, and Tree-fusing parameters. Consistency (CI) and retention (RI) indices were obtained employing the script by Spiekman et al.^22^ that does not take into account a priori deactivated terminals.

## Nomenclatural acts

This published work and the nomenclatural acts it contains have been registered in ZooBank, the online registration system for the International Code of Zoological Nomenclature. The ZooBank LSIDs (Life Science Identifiers) can be resolved and the associated information viewed through any standard web browser by appending the LSID to the prefix ‘http://zoobank.org/’. The LSID for this publication is: urn:lsid:zoobank.org:pub:80B862A6-E984-4AC1-801C-7100CC605BFF.

## Results

Systematic paleontology

Archosauria Cope, 1869

Pseudosuchia Zittel, 1887–1890

Gracilisuchidae Butler et al., 2014

*Parvosuchus aurelioi* gen. et sp. nov.

[urn:lsid:zoobank.org:act:1CA45C46-8AD3-4515-9683-54A7423BE716 (genus)]

[urn:lsid:zoobank.org:act:0D35B9EA-FE83-4DBE-B1BD-AEF7E2CDC223 (species)]

### Holotype

CAPPA/UFSM 0412, a partial skeleton, including a skull with lower jaws, 11 dorsal vertebrae, two sacral vertebrae, a complete pelvic girdle, both femora (lacking the distal portion), partial left tibia, partial left fibula, and left calcaneum.

### Etymology

The genus name combines the Latin word “parvus” (= small) and the Greek word “suchus” (= crocodile). The specific epithet honors Pedro Lucas Porcela Aurélio for his passion for paleontology and prospecting, as well as for having discovered the fossil material described here.

### Type locality, age, and horizon

**Figure 1:**
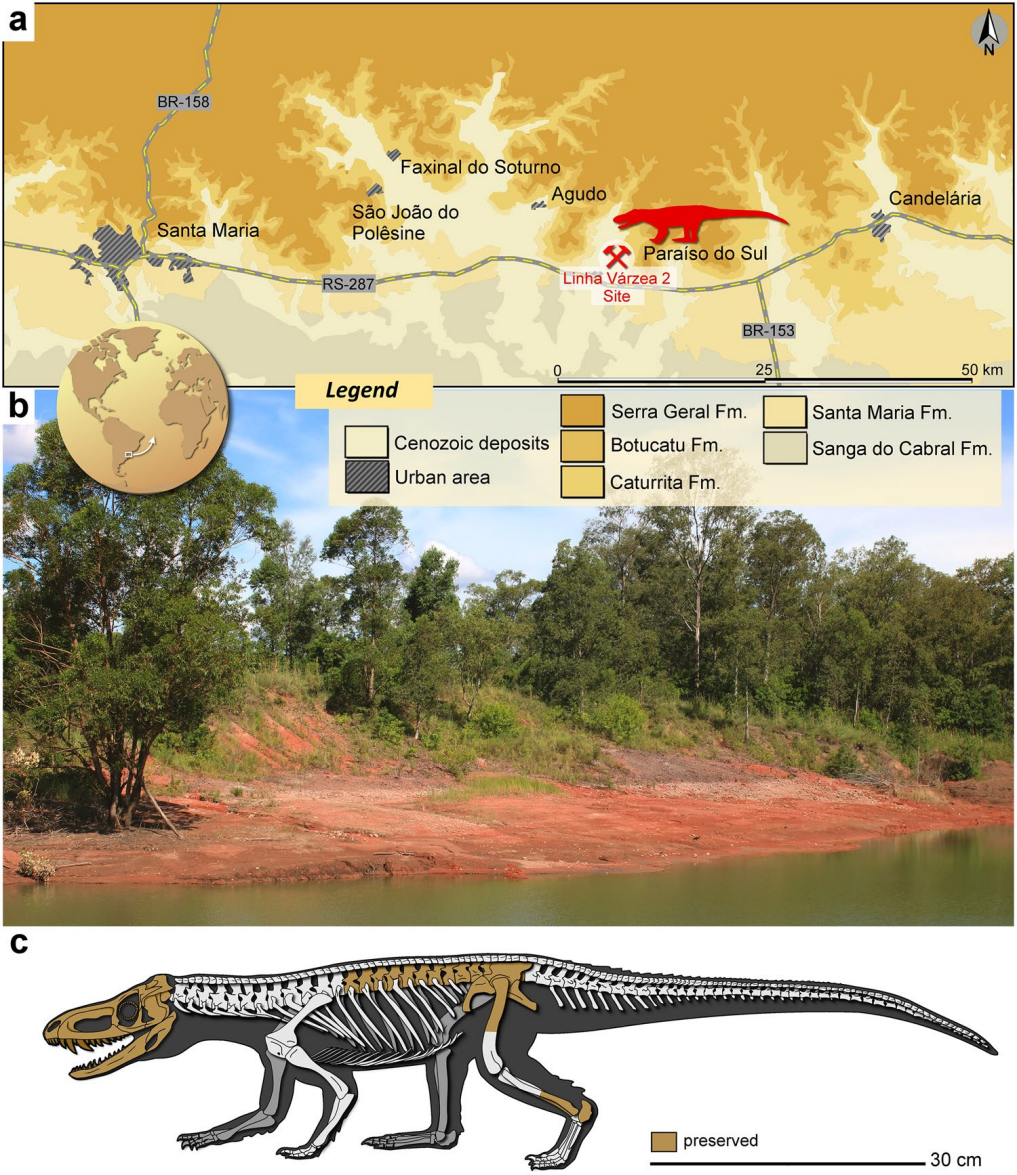
Provenance of *Parvosuchus aurelioi* gen. et sp. nov. (**a**) Location and geological context of the Linha Várzea 2 site, Paraíso do Sul, Rio Grande do Sul, Brazil. (**b**) General view of the Linha Várzea 2 site (taken in January 2023). (**c**) Hypothetical reconstruction of the skeleton of the *Parvosuchus aurelioi* gen. et sp. nov. depicting (in orange) the preserved portions of CAPPA/UFSM 0412. Unpreserved portions are based on the skeletal reconstruction of *Gracilisuchus stipanicicorum* by Jorge González^14^.

Linha Várzea 2 (Becker) site (29°43′03″S, 53°09′07″W), municipalities of Paraíso do Sul, Rio Grande do Sul, Brazil (Fig. 1). This site belongs to the Pinheiros-Chiniquá Sequence^23^ of the Santa Maria Supersequence^24^, Paraná Basin. The fossiliferous content of this locality is associated with the *Dinodontosaurus* Assemblage Zone (AZ)^25^. Recent biostratigraphic studies suggest that this AZ has an age close to the Ladinian/Carnian boundary^3,18,26^.

### Diagnosis

*Parvosuchus aurelioi* differs from all other known gracilisuchids with comparable material in (*local autapomorphies): dorsal margin of the orbit markedly elevated above the skull table*; orbit dorsoventrally taller than long*; orbit shorter anteroposteriorly than the anteroposterior length of the antorbital fenestra; premaxilla lack a narial fossa; posterior portion of the parietal elevated above the skull roof*; parietal with a smooth anteromedial corner of the supratemporal fenestra; the ventral margin of the angular is directed upwards, forming an oblique angle with the ventral margin of the dentary*; craniomandibular joint above maxillary dental margin; and short pubis* with an anteroposterior distal expansion (see the “Discussion” for a differential diagnosis).

### Description

**Figure 2:**
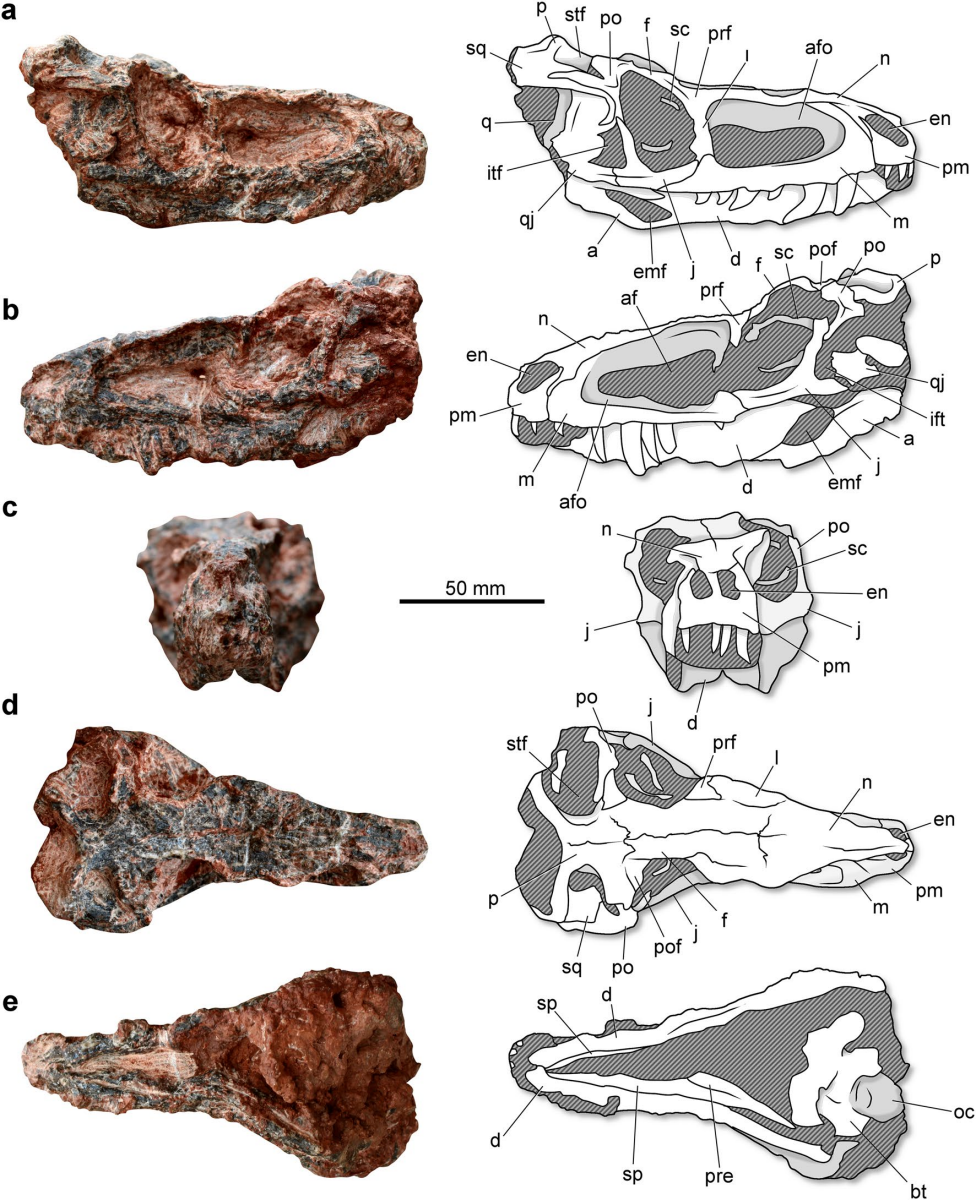
Skull and lower jaws of *Parvosuchus aurelioi* gen. et sp. nov. from the Pinheiros-Chiniquá Sequence (Ladinian-Carnian boundary) of the Santa Maria Supersequence, southern Brazil. Holotype (CAPPA/UFSM 0412) in right lateral (**a**), left lateral (**b**), anterior (**c**), dorsal (**d**), and ventral (**e**) views. a, angular; af, antorbital fenestra; afo, antorbital fossa; bt, basal tubera; d, dentary; emf, external mandibular fenestra; en, external naris; f, frontal; i, infratemporal fenestra; j, jugal; l, lacrimal; m, maxilla; n, nasal; o, occipital condyle; p, parietal; pm, premaxilla; po, postorbital; pof, postfrontal; pre, prearticular; prf, prefrontal; q, quadrato; qj, quadratojugal; sc, sclerotic ring; sp, splenial; sq, squamosal; stf, supratemporal fenestra.

*Parvosuchus aurelioi* was a relatively small pseudosuchian. Its skull (Fig. 2) is 144 mm in length (from the tip of the premaxilla to the occipital condyle). The premaxilla is anteroposteriorly short and bears an elongated posterolateral process that dorsally (Fig. 2a). The external naris is reduced and lack any associated fossa. A fossa occurs in the anteroventral corner of the external naris of *Maehary bonapartei*^16^. The number of tooth positions in the premaxilla is not clear. The alveolar margin of the premaxilla runs on the same plane that of the maxillary alveolar margin (Fig. 2b). The maxilla is elongated and gracile, with a posterior process that forms part of the antorbital fossa. Whereas there is a subtle dorsal expansion on the posterior portion of the posterior process, most of the posterior portion of this process presents a similar dorsoventral depth as the anterior portion ventral to the antorbital fenesta. A well-developed antorbital fossa excavated the ascending process of the maxilla. In lateral view, the ascending process present the width along all its length. Whereas the number of tooth positions is uncertain because of preservational biases, it is possible to note that the teeth reach the posterior portion of the bone. Some of the preserved teeth reveal a posteriorly recurved crown with a pointed apex. The poor preservation hampers the observation of possible serrations. The anterolateral process of the nasal is elongated and runs between the premaxilla and nasal. The bone forms a sharp lateral roof over the antorbital fenestra (Fig. 2c). The main body of the jugal is dorsoventrally tall and expanded transversely, forming a shelf at the ventral part of the orbital margin. There is a longitudinal ridge on the lateral surface of the bone (Fig. 2a,b), which is absent in *Yonghesuchus sangbiensis*^12^. The posterior process of the jugal is anteroposteriorly shorter than the main body, contrasting with *Yonghesuchus sangbiensis*^12^. The lacrimal forms the posterior portion of the dorsal margin of the antorbital fenestra. The orbit occupies approximately 17.3% of the total length of the skull, whereas the external antorbital fenestra comprises 33.7% of the length (Table 1). The orbit is taller than long, contrasting with the orbit of *Gracilisuchus stipanicicorum* and *Turfanosuchus dabanensi* anteroposteriorly, which is anteroposteriorly longer than tall^9–11^. The orbital margin of the frontal is dorsally projected, resulting in an orbital rim that strongly exceeds the dorsal margin of the skull roof. In *Gracilisuchus stipanicicorum*^10^ it is slightly elevated regarding the skull table, whereas in *Turfanosuchus dabanensis*^11^ it is not elevated. A small postorbital forms the posterodorsal corner of the orbital rim. The postorbital forms the main portion of the posterior border of the orbit. Distinct from *Turfanosuchus dabanensis*^11^, the ventral process if not distinctly anteriorly flexed. The squamosal folds over the quadrate head. The anterior portion of the ventral process contacts the posterior margin of the ventral process of the postorbital. As a result, the infratemporal fenestra is dorsoventrally short (Fig. 2a). This condition differs the specimen from the condition observed in *Turfanosuchus dabanensis*^11^, which bears an infratemporal fenestra that is divided in a ventral and dorsal fenestra by an anterior inflection of the squamosal. The supratemporal fenestra is transversely wider than it is anteroposteriorly long (Fig. 2d). The posterior portion of both parietals is elevated above the skull roof. There is a depression on the lateral surface of the main body of the parietal, which forms the medial margin of the supratemporal fossa. Although the bone surface is poorly preserved, it is possible to note that the dorsal surface of the skull is adorned with irregular sculptures on the nasal, frontal, and parietal bones. The holotype of *Parvosuchus aurelioi* preserves some ossicles within both orbital cavities, which are interpreted as parts of the slender sclerotic rings. The ring diameter is approximately 18 mm and the ossicles are approximately 2 mm in height.

**Table 1:** Measurements (in mm) of the skull of *Parvosuchus aurelioi* gen. et sp. nov. (CAPPA/UFSM 0412).

Dimension	Measurement
Skull length	144
Preorbital skull length	79
Maximum diameter of external naris	16
Minimum interorbital width	21
Maximum temporal width	73
Vertical diameter of orbit	34
Anteroposterior diameter of orbit	25
Maximum length of antorbital fenestra	49
Length of lower jaw	124

The lower jaws are not entirely observable because the mandible is in occlusion. The mandibular symphysis is restricted to the anterior tip of the dentary, lacking any participation of the splenial (Fig. 2e). The slender dentary comprises the main portion of the lower jaw in lateral view. The bone reaches the level of the posterior half of the orbit and forms the anterior margin of the external mandibular fenestra (Fig. 2b). The posterior portion is composed of two processes. The ventral process is longer and contributes to the anterior half of the dorsal margin of the external mandibular fenestra. The presence of a surangular shelf is uncertain. In lateral view, the ventral margin of the angular is directed upwards (Fig. 2b), forming an oblique angle with the ventral margin of the dentary. In *Gracilisuchus stipanicicorum*^10^ and *Yonghesuchus sangbiensis*^12^ the ventral margin of the angular runs in the same longitudinal plane as the ventral margin of the dentary. The craniomandibular joint is just slightly above the maxillary dental margin.

**Figure 3:**
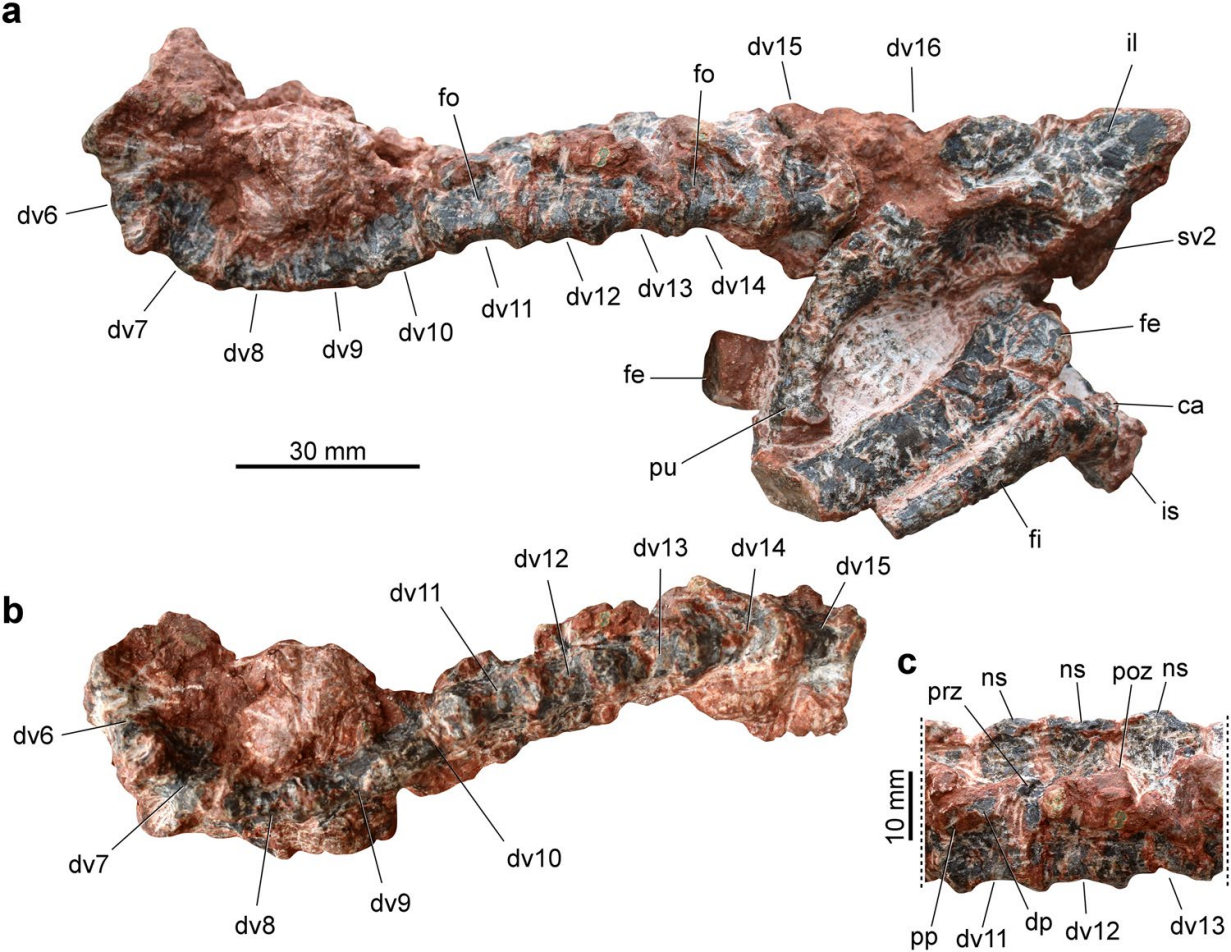
Axial skeleton of *Parvosuchus aurelioi* gen. et sp. nov. from the Pinheiros-Chiniquá Sequence (Ladinian-Carnian boundary) of the Santa Maria Supersequence, southern Brazil. Dorsal vertebrae of the holotype (CAPPA/UFSM 0412) in left lateral (**a**) and ventral (**b**) views. (**c**) Dorsal vertebrae 11 to 13 in left lateral view. ca, calcaneum; dp, diapophysis; dv, dorsal vertebra; fe, femur; fi, fibula; fo, fossa; il, ilium; is, ischium; ns, neural spine; poz, postzygapophysis; pp, parapophysis; prz, prezygapophysis; pu, pubis; sv, sacral vertebra.

**Figure 4:**
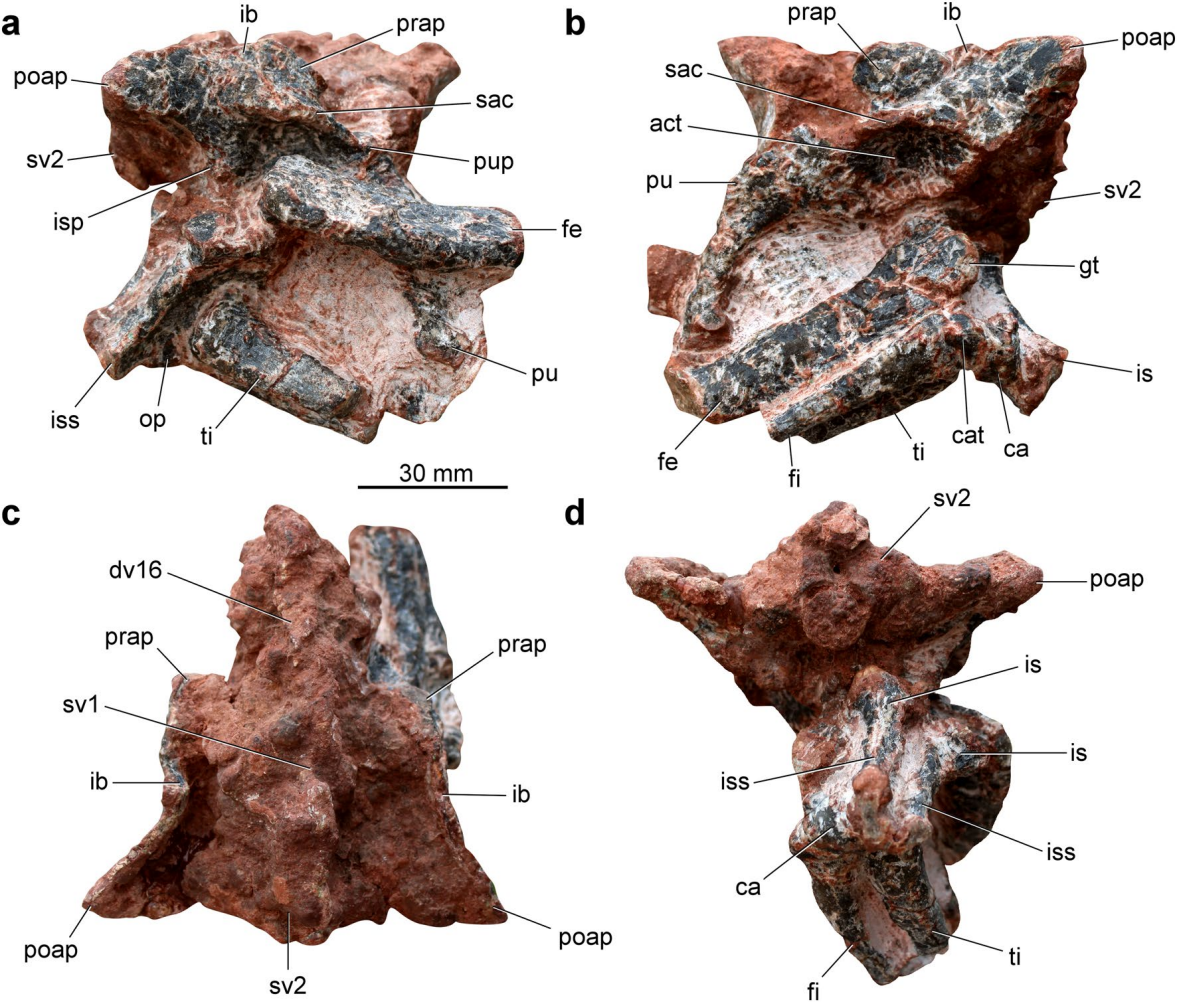
Pelvic girdle and hindlimb of *Parvosuchus aurelioi* gen. et sp. nov. from the Pinheiros-Chiniquá Sequence (Ladinian-Carnian boundary) of the Santa Maria Supersequence, southern Brazil. Pelvic girdle of the holotype (CAPPA/UFSM 0412) in right lateral (**a**), left lateral (**b**), dorsal (**c**), and ventral (**d**) views. act, acetabulum; ca, calcaneum, cat, calcaneal tuber; dv, dorsal vertebra; fi, fibula; fe, femur; gt, greater trochanter of the femur; ib, iliac blade; is, ischium; isp ischiadic peduncle of the ilium; iss, ischial shaft; op, obturator plate of the ischium; poap, postacetabular process; prap, preacetabular process; pu, pubis; pup, pubic peduncle of the ilium; sac, supraacetabular crest; sv, sacral vertebra.

The holotype of *Parvosuchus aurelioi* preserves a series of 13 articulated vertebrae, which includes 11 dorsal (6 to 16 dorsal elements) and the two sacral vertebrae (Fig. 3). The vertebral centra are spool-shaped and increase in length from the middle to the posterior elements (Table 2). There is shallow fossa that is not rimmed on the lateral surface of each centrum (Fig. 2a). The shape of the articular structures is poorly preserved. The anterior tip of the prezygapophysis barely extends beyond the anterior margin of their respective centrum. The parapophysis is situated on the neural arch in the observable elements. The transverse process is moderately expanded laterally. Neural spine of the posterior dorsal vertebrae is subrectangular and is slightly longer than tall. Furthermore, the spines lack a distal expansion. There are no neurocentral sutures. On the other hand, the bone surface of the specimen is poorly preserved. Therefore, this condition show be considered with carefully. The two sacral vertebrae are covered by a thick layer of concretion, hampering the observation of fine details. Nevertheless, it is safe to say that the sacrum is composed solely of two vertebrae (Fig. 4c). The neural spine of both vertebrae lacks a distal expansion. The sacral rib of the second vertebra expands anteroposteriorly at its distal end. The neural arch of the second sacral vertebra seems not fused to their respective centrum (Fig. 4d).

**Table 2:** Measurements (in mm) of the axial skeleton of *Parvosuchus aurelioi* gen. et sp. nov. (CAPPA/UFSM 0412).

Vertebra	Centrum length	Centrum height (posterior)	Neural spine height
Dorsal vertebra 6	14	10	–
Dorsal vertebra 7	13	9	–
Dorsal vertebra 8	15	10	–
Dorsal vertebra 9	15	11	–
Dorsal vertebra 10	15	10	12
Dorsal vertebra 11	14	10	13
Dorsal vertebra 12	15	11	12
Dorsal vertebra 13	16	11	12
Dorsal vertebra 14	16	11	–
Dorsal vertebra 15	16	11	12
Dorsal vertebra 16	–	–	–
Sacral vertebra 1	–	–	10
Sacral vertebra 2	17	10	10

The ilium is short (Table 3), with approximately 53 mm in length. It comprises approximately 36.5% the total length of the skull. The preacetabular process is short and the anterior tip is rounded to sub-rectangular (Fig. 4b). There is no a vertical ridge posterior to the preacetabular process. The postacetabular process is elongated, with a posterior rounded to triangular margin (Fig. 4a). The main axis of the postacetabular process directs laterally along its posterior portion (Fig. 4c). The dorsal margin of the iliac blade is straight, such as in *Turfanosuchus dabanensis*^12^, whereas in *Gracilisuchus stipanicicorum*^13^ it is convex. The iliac blade is relatively low, as a result, the dorsal portion of the ilium is about the same height as the portion ventral to the supracetabular crest (i.e., acetabular region). The supracetabular crest rises from the lateral surface of the pubic peduncle and extends posteriorly, roofing the acetabulum. The crest smoothly merges with the main body of the bone dorsal the posterior half of the acetabulum. The ischiadic peduncle is short and lacks a posterior heel. In lateral view, the posterior transition between the ischiadic peduncle and the postacetabular process is concave. The medial wall of the iliac acetabulum is well-developed and convex ventrally.

**Table 3:** Measurements (in mm) of the pelvic girdle and hindlimb of *Parvosuchus aurelioi* gen. et sp. nov. (CAPPA/UFSM 0412).

Dimension	Measurement
Ilium	
Blade length	45
Pubic peduncle length	10
Acetabulum, anteroposterior diameter	23
Ischium	
Length	41
Transverse shaft diameter	4
Pubis	
Length	43
Blade, midlength transverse width	9
Femur	
Head, maximum width	23
Midshaft, maximum width	13
Midshaft, minimum width	9
Tibia	
Distal end, maximum anteroposterior length	14
Fibula	
Distal end, maximum anteroposterior length	13

The pubis is short (Table 3; approximately 43 mm in length) and anteroventrally oriented (Fig. 4b). There is a tuber on the anterolateral surface of the proximal portion. The iliac peduncle is anteroposteriorly elongated. The contribution of the pubis to the acetabulum is minimal do absent. The medial portion of the pubic apron is poorly preserved. There is a subtle anteroposterior expansion on the distal end of the pubis. The ischium is 41 mm in length (Table 3). The outline of the iliac peduncle is triangular and faces dorsally. An acetabular margin expands laterally (Fig. 4a). The contact with the pubis is not clear. The ischial shaft contacts its counterpart along its entire length. Conversely, there is no contact between the dorsal portion of the ischial shafts (Fig. 4d). As a result, there is a wide longitudinal groove between both ischia. The shaft is plate-like in cross-section. An obturator plate projects ventrally from the main body, extending along the shaft.

The total length of the hindlimb elements is uncertain because these bones are not entirely preserved. The proximal half of both femora is preserved. Conversely, there are no remains of the distal portion. The femoral head is slightly expanded in the anteroposterior axis (23 mm). It lacks any feature between the ventral transition from the head to the femoral shaft, resulting in a smooth transition (Fig. 4b). There is a straight and anteroposteriorly oriented groove on the proximal end, which is absent in *Turfanosuchus dabanensis*^11^. The anterolateral tuber is rounded and connects to a ridge that runs distally on the lateral surface of the femoral head. This longitudinal ridge bounds the anterior margin of a depression that extends along the posterior half of the lateral surface of the femoral head. The posteromedial tuber is well-developed. The proximal surface lacks any evidence of a trochanteric fossa. The greater trochanter is rounded. The proximal portion of the bone lacks an anterior (= lesser) trochanter and a trochanteric shelf. The mid-shaft is elliptical (Table 3), with the main axis anteroposteriorly oriented (13 mm axis anteroposterior and 9 mm axis lateromedial). The shape of the fourth trochanter is obscured by the matrix.

The specimen preserves the distal portions of the left tibia (Fig. 4a) and fibula (Fig. 4b), as well as the left calcaneum (Fig. 4b). The tibial shaft is stouter than that of the fibula. It is elliptical in cross-section. There is a longitudinal ridge on the anteromedial corner of the shaft. The tibia expands at its distal portion (Table 3), with the main axis of the expansion being anteroposteriorly oriented (14 mm). The anteromedial corner of the distal end of the tibia is rounded. In medial view, the anterior portion of the distal end is more distally projected than the posterior portion. Conversely, the posterior corner of the distal end expands posteriorly, resulting in a slightly concave posterior margin of the shaft. The fibular shaft is slender, expanding gradually towards the distal portion (Table 3). No ridges or tubers are present on the preserved portion of the shaft. The distal end of the fibula bends medially. The calcaneum is partially obscured by the fibula and ischia. The bone is anteroposteriorly elongated and bears a conspicuous calcaneal tuber. In lateral view, there is a marked notch between the calcaneal tuber and the calcaneal condyle. The latter forms the anterior portion of the bone. The posterior portion of the calcaneal tuber is upward directed. There is a fossa on the ventral/distal surface of the tuber.

### Phylogenetic analysis

**Figure 5:**
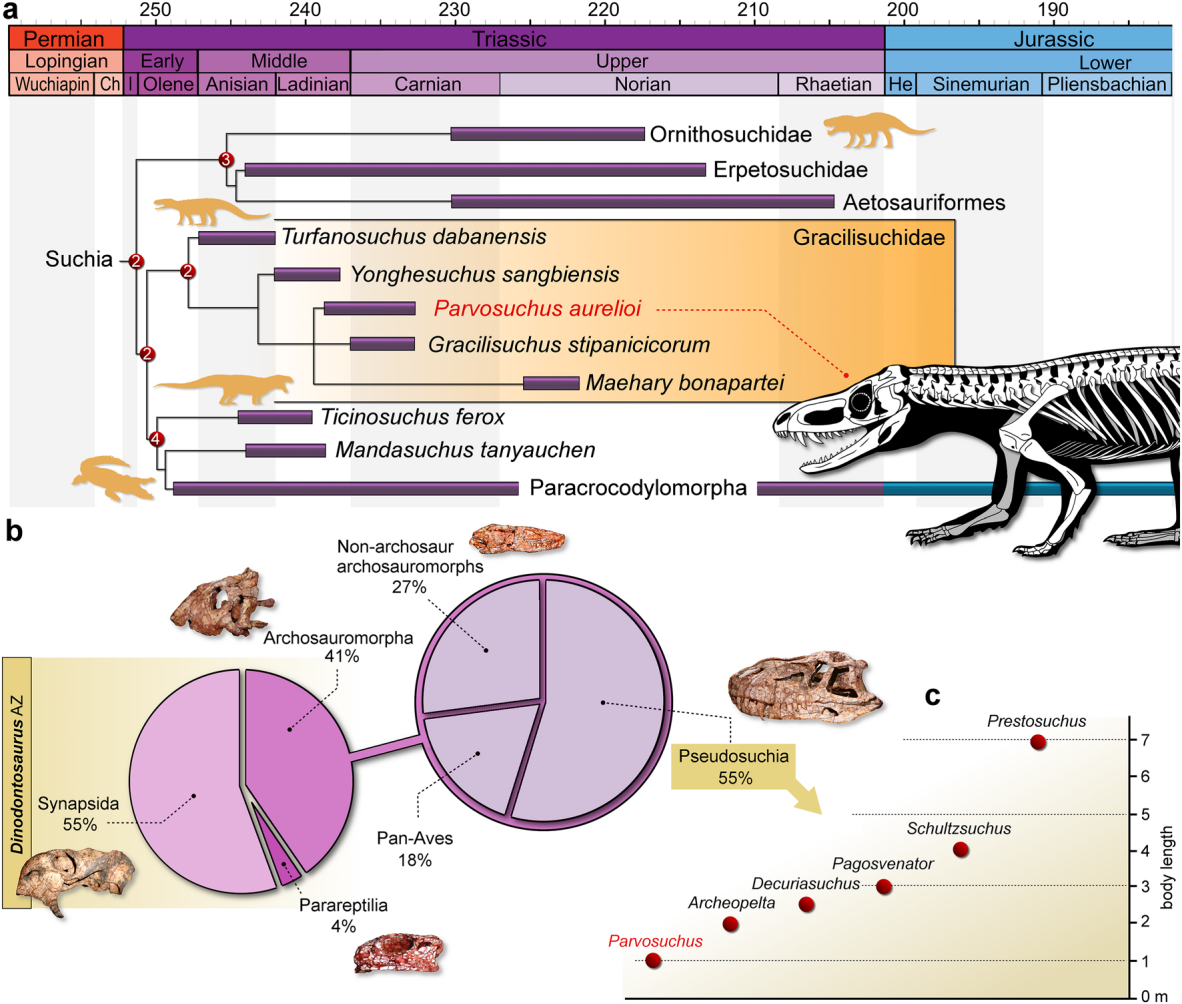
Results of the phylogenetic analysis and diversity of the *Dinodontosaurus* Assemblage Zone of Brazil. (**a**) Time-calibrated reduced strict consensus tree depicting the phylogenetic position of *Parvosuchus aurelioi* gen. et sp. nov. Number on nodes represent Bremer support values higher than 1. The temporal bars for each OTU represent the maximum and minimum ages of each geological unit. Divergence times set as approximately 1 million years. (**b**) Percentage of taxonomic groups recorded in the *Dinodontosaurus* Assemblage Zone according to the number of species. (**c**) Approximate body length of pseudosuchian species from the *Dinodontosaurus* ZA.

The analysis recovered 54,000 MPTs of 6773 steps each (CI = 0.17437; RI = 0.65586). In the strict consensus tree, *Parvosuchus aurelioi* is found as a member of Gracilisuchidae, within an unsolved node supporting *Gracilisuchus stipanicicorum* and *Maehary bonapartei* (Fig. 5a). This node is supported by: the ascending process of the maxilla with the same width along all its length in lateral view (ch. 60: 0 → 1); and posterior process of the maxilla with a similar dorsoventral depth as the anterior portion ventral to the antorbital fenestra (ch. 63: 0 → 1). *Turfanosuchus dabanensis* is the basalmost member of Gracilisuchidae. The clade is supported by 11 synapomorphies. Regarding the inner affinities of Gracilisuchidae, *Yonghesuchus sangbiensis* is the sister taxon of the node supporting *Parvosuchus aurelioi*, *Gracilisuchus stipanicicorum* and *Maehary bonapartei*. An interesting point comprises the position of *Maehary bonapartei*, which was not affected by the inclusion of *Parvosuchus aurelioi*. Therefore, the present analysis provides further support for including *Maehary bonapartei* in Gracilisuchidae^2^ rather than Pterosauromorpha^16^. Regarding the general topology recovered in the present analysis, the strict consensus tree maintains the same topology as that presented by Müller et al.^2^.

## Discussion

*Parvosuchus aurelioi* is a lightly built pseudosuchian from the clade Gracilisuchidae. In addition to the phylogenetic analysis performed here, a series of typical traits of gracilisuchids are present in the holotype, reinforcing this assignation, such as: an enlarged antorbital fenestra; triangular expansion of the dorsal margin of the posterior process of the maxilla; expanded lateral margin of the nasal, forming a lateral roof; and size of the infratemporal fenestra is reduced due to the anterior expansion of the quadratojugal. Moreover, the new gracilisuchid differs from all the other members of the clade by a series of traits, as follows below.

*Turfanosuchus dabanensis* differs from *Parvosuchus aurelioi* in that the former has: dorsal margin of the orbit level with skull table or raised slightly; orbit dorsoventrally anteroposteriorly longer than tall; anterior portion of the nasal elevated above skull roof in lateral view; squamosal with an elongated ventral process; infratemporal fenestra divided into two fenestrae; posterior portion of the parietal not elevated above the skull roof; quadrate head partially exposed laterally; well-developed posteroventral process of the dentary; and longer pubis.

*Gracilisuchus stipanicicorum* differs from *Parvosuchus aurelioi* in that the former has: orbit anteroposteriorly longer than tall; orbit anteroposteriorly longer than the antorbital fenestra; slightly longer posterior process of the jugal; parietal extends over the interorbital region; parietal with a sinuous shelf on the anteromedial corner of the supratemporal fenestra; posterior portion of the parietal not elevated above the skull roof; ventral margin of the angular runs in the same longitudinal plane as the ventral margin of the dentary; craniomedial joint at the same level as the dental margin of the maxilla; convex dorsal margin of the iliac blade of the ilium; longer pubis; and pubis lack an anteroposterior distal expansion.

*Yonghesuchus sangbiensis* differs from *Parvosuchus aurelioi* in that the former has: elongated anterior portion of the maxilla; lack a ridge or bump on the lateral surface of the jugal; posterior process of the jugal is approximately half the total length of the bone (it is approximately 0.25 in *Parvosuchus aurelioi*); ventral margin of the angular runs in the same longitudinal plane as the ventral margin of the dentary; and craniomedial joint at the same level as the dental margin of the maxilla.

*Maehary bonapartei* differs from *Parvosuchus aurelioi* in that the former has: premaxilla with an expanded narial fossa in the anteroventral corner of the naris; lateral margin of the nasal is poorly developed, not forming a lateral shelf; and distal edge of the maxillary tooth crowns is straight or gently sigmoidin in labial or lingual views.

The inclusion of *Parvosuchus aurelioi* in the phylogenetic analysis did not affect the placement of Gracilisuchidae within the phylogenetic tree of Pseudosuchia, reinforcing the hypothesis that gracilisuchids are an early diverging clade of suchians. This same arrangement has been recovered in previous iterations of the present data matrix^2,4,16,18,27^, including through Bayesian phylogenetic analysis^2,4^. The present analysis also corroborates that gracilisuchids are more closely related to paracrocodylomorphs than to Ornithosuchidae, Erpetosuchidae, or Aetosauriforms.

Given that other gracilisuchids have not been identified within the Santa Maria Formation^20^, it is unlikely that *Parvosuchus aurelioi* represents a distinct ontogenetic stage of a known species. On the other hand, there is an enigmatic archosauromorph named *Barberenasuchus brasiliensis*^28^ from the same geological unit that yielded *Parvosuchus aurelioi*. *Barberenasuchus brasiliensis* is considered an indeterminate archosauriform with carnivorous feeding habits^29^. This archosauriform is particularly interesting because it is a small-sized animal (skull with less than 100 mm in length), representing one of the few records of a small reptile with predatory behavior from the *Dinodontosaurus* AZ. Conversely, the holotype (MCP-PV 220), the only known specimen, is poorly preserved, hampering properly systematic assignations^29,30^. Different interpretations have been proposed regarding the affinities of this taxon, such as a crocodylomorph^31^ or a dinosauromorph^32^. While it has not been classified as a gracilisuchid thus far, it is important to note that *Barberenasuchus brasiliensis* differs from *Parvosuchus aurelioi* in that the former has: dorsal margin of the orbit level with skull table; orbit anteroposteriorly longer than the antorbital fenestra; longer anterior process of the maxilla; dorsal end of the ascending process of the maxilla does not extend posteriorly to form the dorsal margin of the antorbital fossa; antorbital fossa has participation of the anterior process of the jugal; posterior margin of the dorsal portion of the parietal lies below the dorsal margin of the skull table; infratemporal is taller (taller than half of the orbital height); supratemporal fenestra anteroposteriorly longer than wide; lateral tip of the paraoccipital process exceed the lateral margin of the supratemporal fenestra in dorsal view; ventral margin of the angular runs in the same longitudinal plane as the ventral margin of the dentary; and craniomedial joint at the same level as the dental margin of the maxilla.

**Figure 6:**
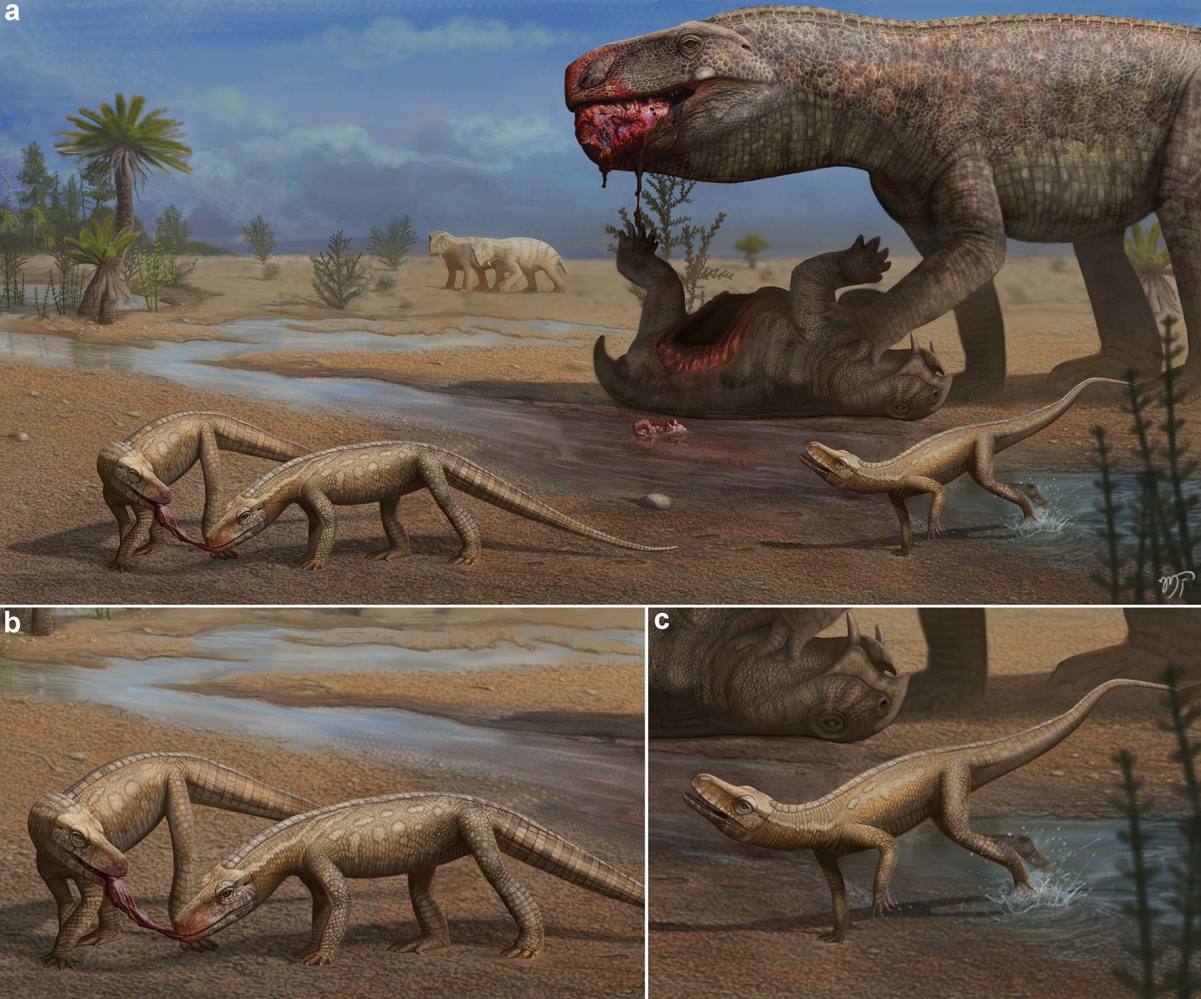
Artistic representation of a Middle-Late Triassic landscape of southern Brazil. (**a**) A large *Prestosuchus chiniquensis* feeds on the carcass of a dicynodont while individuals of *Parvosuchus aurelioi* gen. et sp. nov. compete for scraps. (**b**) and (**c**) depict details of individuals of *Parvosuchus aurelioi* gen. et sp. nov. Artwork by Matheus Fernandes.

The Triassic ecosystems that preceded the radiation of dinosaurs were composed of a wide variety of reptiles from the crocodilian lineage^18^. This diversity is well documented in fossiliferous layers of the Chañares Formation in Argentina^18^ and Santa Maria Formation in Brazil^20^. New representatives from different groups of pseudosuchians have been reported in recent years from the Santa Maria Formation^33,34^. As a result, the diversity of pseudosuchian archosaurs has been increasing, but some groups that occur in the Chañares Formation had not yet been reported for Brazil. The discovery of *Parvosuchus aurelioi* fills the gap regarding the Gracilisuchidae. In addition to expanding the taxonomic diversity of pseudosuchians from the *Dinodontosaurus* AZ, the new species also provides important contributions to the understanding of the paleofaunal composition that existed in southern Brazil during the Ladinian to early Carnian. Represented by cynodonts^35^ and dicynodonts^36^, synapsids encompass the majority of the diversity within the *Dinodontosaurus* AZ^20^ (Fig. 5b). Whereas these dicynodonts are strictly large and herbivorous^36^, the record of cynodonts includes a certain ecomorphological diversity, encompassing carnivorous to omnivorous/herbivorous forms^35^. Far less abundant and diverse is the record of parareptiles, represented by the tiny *Candelaria barbouri*^37,38^, a owenettid procolophonoid. Archosauromorpha is relatively diverse, the clade is represented by herbivorous rhynchosaurus^39^, carnivorous proterochampsids^40^, and members of the clade Archosauria^6,33,41,42^. Actually, there is the problematic *Barberenasuchus brasiliensis*^28–30^, which was discussed above. Regarding the record of archosaurs, it is composed of both Pan-Aves and Pseudosuchia. Pan-Aves are represented by the aphanosaurian *Spondylosoma absconditum*^43^, the silesaur *Gamatavus antiquus*^42^*,* and an indeterminate dinosauromorph^26^. According to the holotype, *Spondylosoma absconditum* was carnivorous and reached approximately 1.75 m in length. *Gamatavus antiquus* was approximately 1.5 m in length and its feeding behaviors are uncertain. Whereas there are no teeth referred to this silesaur^42^, most members of this group are considered omnivorous or herbivorous^44^. Pseudosuchians are more diverse compared to Pan-Aves in the *Dinodontosaurus* AZ^20^. The clade is represented by *Archeopelta arborensis*^45^, *Pagosvenator candelariensis*^41^*, Schultzsuchus loricarus*^34^, *Prestosuchus chiniquensis*^6,7^*,* and *Decuriasuchus quartacolonia*^33^. *Archeopelta arborensis* and *Pagosvenator candelariensis* were carnivorous members of Erpetosuchidae^18,27,41^, both reached more than 2 m in length. *Schultzsuchus loricarus* was a large carnivorous from the clade Poposauroidea^34^. Finally, *Prestosuchus chiniquensis* and *Decuriasuchus quartacolonia* were carnivorous loricatans. Some recent histological analysis suggested that the specimens of *Decuriasuchus quartacolonia* were juvenile organisms^46^. Because these specimens are recorded from the same fossiliferous site that yielded specimens of *Prestosuchus chiniquensis*, it is plausible that they represent early ontogenetic stages of this large loricatan. Nevertheless, even the specimens of *Decuriasuchus quartacolonia* are not small animals, reaching approximately 2.5 m in length. The discovery of *Parvosuchus aurelioi* expands the diversity of pseudosuchians from the *Dinodontosaurus* AZ and represents the smallest pseudosuchian from this AZ (Fig. 5c). Bearing a skull with a length of 144 mm, *Parvosuchus aurelioi* is estimated to have reached approximately 1 m in body length based on comparisons with other related pseudosuchians. The inclusion of this new pseudosuchian in the paleofauna of the *Dinodontosaurus* AZ sheds light on the composition of terrestrial ecosystems that preceded the origin of dinosaurs in southern Brazil. The elongated and blade-like teeth of *Parvosuchus aurelioi* are typically associated with carnivorous feeding habits. Therefore, it represents the first small-sized predatory pseudosuchian from an environment dominated by huge archosaur predators (Fig. 6).

The presence of a gracilisuchid in the *Dinodontosaurus* AZ reinforces the biostratigraphic similarity between this AZ and the Chañares Formation in Argentina. Nevertheless, the Chanãres Formation encompasses two distinct AZs, the *Massetognathus-Chanaresuchus* AZ and the *Tarjadia* AZ^18^. The *Massetognathus-Chanaresuchus* AZ overlaps the *Tarjadia* AZ and is early Carnian in age according to radioisotopic datings^47^, whereas the *Tarjadia* AZ is assigned to the Ladinian-Carnian boundary^18^. The shared presence of phylogenetic related erpetosuchids, rhynchosaurs, suchians, and cynodonts between the *Massetognathus-Chanaresuchus* AZ and the *Dinodontosaurus* AZ provided support for biostratigraphic proposals correlating both AZs^3,18,20,26^. On the other hand, the record of some groups of cynodonts^35^, dinosauromorphs^42^, and proterochampsids^40^ in the *Dinodontosaurus* AZ resembles more the fossil content of the *Massetognathus-Chanaresuchus* AZ. Similarly, whereas gracilisuchids are absent in the *Tarjadia* AZ, the clade occurs in the *Massetognathus-Chanaresuchus* AZ^18^. Moreover, radioisotopic datings suggested a maximum depositional age of 237 Ma (early Carnian) to the Brazilian *Santacruzodon* AZ^48^. This AZ overlaps the *Dinodontosaurus* AZ^20,23^, suggesting that it is more likely associated with the Ladinian-Carnian boundary or even older. Therefore, the occurrence of the gracilisuchid *Parvosuchus aurelioi* in the *Dinodontosaurus* AZ emphasizes the need for refinement in recognizing the different paleofaunas commonly assigned to the *Dinodontosaurus* AZ.

## Conclusions

The new specimen described here bears a unique set of traits that led to the naming of a new pesudosuchian taxon. *Parvosuchus aurelioi* is the first Brazilian gracilisuchid found in layers attributed to the Ladinian-Carnian boundary. The inclusion of a small carnivorous pseudosuchian in the paleofauna of the *Dinodontosaurus* AZ expands our knowledge regarding the faunal composition of this biostratigraphic unit. Lastly, the occurrence of gracilisuchids in the same AZ that contains large paracrocodylomorphs, erpetosuchids, and rhynchosaurs demonstrates a complex biostratigraphic context, suggesting that the *Dinodontosaurus* AZ may contain subdivisions that have not yet been recognized.

### Supplementary Information

Supplementary Information.

## Data Availability

All data generated or analysed during this study are included in this published article and its supplementary information files.
